# DNA Methylation Alterations in Fractionally Irradiated Rats and Breast Cancer Patients Receiving Radiotherapy

**DOI:** 10.3390/ijms232416214

**Published:** 2022-12-19

**Authors:** Magy Sallam, Mohamed Mysara, Mohammed Abderrafi Benotmane, Radia Tamarat, Susana Constantino Rosa Santos, Anne P. G. Crijns, Daan Spoor, Filip Van Nieuwerburgh, Dieter Deforce, Sarah Baatout, Pieter-Jan Guns, An Aerts, Raghda Ramadan

**Affiliations:** 1 Radiobiology Unit, Interdisciplinary Biosciences, Belgian Nuclear Research Centre, SCK CEN, 2400 Mol, Belgium; magy.sallam@sckcen.be (M.S.); mmaysara@nu.edu.eg (M.M.); rafi.benotmane@sckcen.be (M.A.B.); sarah.baatout@sckcen.be (S.B.); an.devries.aerts@gmail.com (A.A.); 2 Laboratory of Physiopharmacology, University of Antwerp, 2610 Wilrijk, Belgium; pieter-jan.guns@uantwerpen.be; 3 Institut de Radioprotection et de Sureté Nucléaire (IRSN), PRP-HOM, SRBE, LR2I, 92260 Fontenay-aux-Roses, France; radia.tamarat@irsn.fr; 4 Centro Cardiovascular da Universidade de Lisboa (CCUL@RISE), Lisbon School of Medicine of the Universidade de Lisboa, 1649-028 Lisbon, Portugal; sconstantino@medicina.ulisboa.pt; 5 Department of Radiation Oncology, University Medical Center Groningen, University of Groningen, 9713 GZ Groningen, The Netherlands; a.p.g.crijns@umcg.nl (A.P.G.C.); d.s.spoor@umcg.nl (D.S.); 6 Laboratory of Pharmaceutical Biotechnology, Ghent University, 9000 Ghent, Belgium; filip.vannieuwerburgh@ugent.be (F.V.N.); dieter.deforce@ugent.be (D.D.); 7 Department of Molecular Biotechnology, Ghent University, 9000 Ghent, Belgium

**Keywords:** DNA methylation, gene expression, cardiovascular disease, ionizing radiation, breast cancer patient

## Abstract

Radiation-Induced CardioVascular Disease (RICVD) is an important concern in thoracic radiotherapy with complex underlying pathophysiology. Recently, we proposed DNA methylation as a possible mechanism contributing to RICVD. The current study investigates DNA methylation in heart-irradiated rats and radiotherapy-treated breast cancer (BC) patients. Rats received fractionated whole heart X-irradiation (0, 0.92, 6.9 and 27.6 Gy total doses) and blood was collected after 1.5, 3, 7 and 12 months. Global and gene-specific methylation of the samples were evaluated; and gene expression of selected differentially methylated regions (DMRs) was validated in rat and BC patient blood. In rats receiving an absorbed dose of 27.6 Gy, DNA methylation alterations were detected up to 7 months with differential expression of cardiac-relevant DMRs. Of those, *SLMAP* showed increased expression at 1.5 months, which correlated with hypomethylation. Furthermore, *E2F6* inversely correlated with a decreased global longitudinal strain. In BC patients, *E2F6* and *SLMAP* exhibited differential expression directly and 6 months after radiotherapy, respectively. This study describes a systemic radiation fingerprint at the DNA methylation level, elucidating a possible association of DNA methylation to RICVD pathophysiology, to be validated in future mechanistic studies.

## 1. Introduction

Thoracic radiotherapy has been shown to increase the risk of cardiac toxicity in cancer patients [1,2,3]. Despite the current radiation-sparing techniques, which limit cardiac exposure, radiation-induced cardiovascular disease (RICVD) is still a primary clinical concern that manifests mainly as coronary heart disease, remaining asymptomatic until 10 to 15 years after radiotherapy [4]. However, a 6% decrease in global longitudinal strain (GLS), an early sign of subclinical left ventricular dysfunction, has been reported in breast cancer (BC) patients as early as 6 months after radiotherapy [5,6]. Consequently, investigating early molecular changes in the cardiovascular system after radiotherapy could identify novel, unexamined players in RICVD pathology and/or potential biomarkers to identify patients at risk, thereby allowing earlier countermeasures.

DNA methylation is an epigenetic process essential for development and maintenance of cellular homeostasis, normally associated with transcriptional silencing when affecting gene promoters [7,8]. Alterations in DNA methylation have been reported in many diseases, including neurodegenerative diseases such as Parkinson’s disease [9], diabetes mellitus [10] and cancer [11]. In addition, recent research indicates a connection between DNA methylation and cardiovascular disease risk [12,13] with methylation alterations preceding histologically evident atherosclerosis [14]. DNA methylation has been hypothesized to affect atherosclerosis pathogenesis by regulating oxidative stress, inflammation and vascular smooth muscle cell (VSMC) phenotype [15]. Interestingly, radiation has also been shown to affect DNA methylation. Both radiation-induced global hypomethylation, as well as gene-specific hypermethylation, have been reported [4,16,17]. However, the contribution of DNA methylation in X-irradiation-induced cardiac toxicity is underexplored. Consequently, the current study aims to investigate the effects of ionizing radiation on DNA methylation in the blood of irradiated rats and BC patients to provide more clarity on the involvement of DNA methylation in RICVD.

The current study is part of the Horizon 2020 project MEDIRAD (http://www.medirad-project.eu, accessed on 30 June 2021) which addresses the implications of medical low-dose radiation exposure [18]. Within MEDIRAD, the effect of radiation on cardiac dysfunction is investigated using preclinical and clinical experimental models [19,20]. In the current study, we assess the methylation profile of irradiated rats of the preclinical model, with special focus on cardiac-relevant differentially methylated regions (DMRs). We also investigate the expression profile of the cardiac-relevant rat-identified DMRs in 25 BC patients from the MEDIRAD EARLY-HEART cohort, the latter cohort being a European multicenter study involving 250 BC patients treated with adjuvant radiotherapy and followed up for 2 years after initial treatment [20].

## 2. Results

### 2.1. Global Hypomethylation Observed at 12 Months after Whole Heart Rat Irradiation

**Figure 1:**
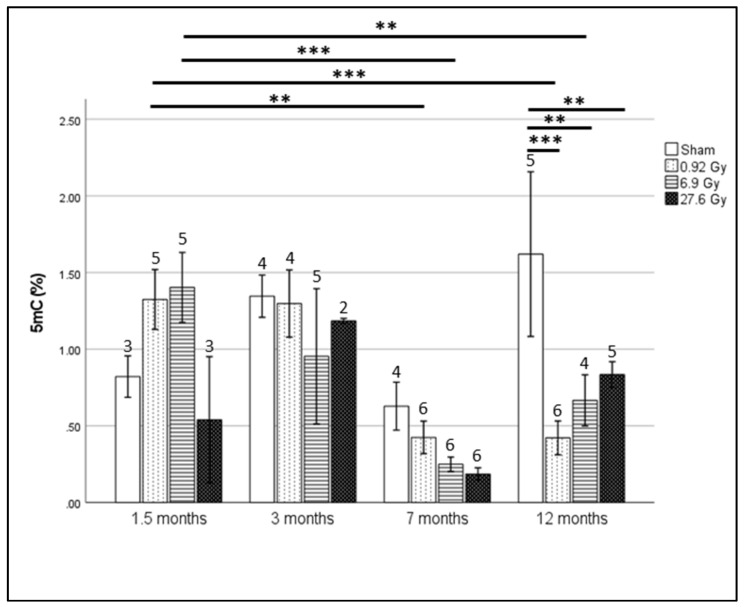
Percentage of 5 mC (%) after fractionated irradiation of 0, 0.04, 0.3 and 1.2 Gy resulting in total irradiation dose of 0, 0.92, 6.9 and 27.6 Gy as measured by MethylFlash Global DNA Methylation (5 mC) ELISA Easy Kit at 1.5, 3, 7 and 12 months after irradiation. Plotted values represent group means ± standard error of mean (SEM) with the number of rats per group indicated per bar. Statistical analysis was performed using SPSS generalized linear model module and multiple comparison correction was performed using least significant difference (LSD) (** = *p*-value < 0.01, *** < 0.001).

The percentage of 5-methyl cytosine (5 mC%) in rat blood DNA after the different irradiation doses at the four sampling time points is shown in Figure 1. The irradiated rats exhibited dose-dependent reduction in global longitudinal strain (GLS) (>15%), as measured by echocardiography, at 12 and 18 months, along with decreased cardiac apex microvascular density after 27.6 Gy [19]. Significant hypomethylation was observed at 12 months after all irradiation doses relative to sham-irradiated rats. This is especially evident in rats irradiated with 0.92 and 6.9 Gy with significantly lower methylation levels observed at 7 and 12 months relative to 1.5 months. Global hypomethylation, as measured by 5 mC%, strongly correlated with the GLS of rats receiving 6.9 and 27.6 Gy at 12 months after fractionated irradiation (FI) (r = |0.998|, *p*-value < 0.05 and r = |0.884|, *p*-value = 0.12, respectively; Supplementary Figure S1).

### 2.2. Gene-Specific DNA Methylation Analysis and Enriched Pathways of Rat DMRs

**Figure 2:**
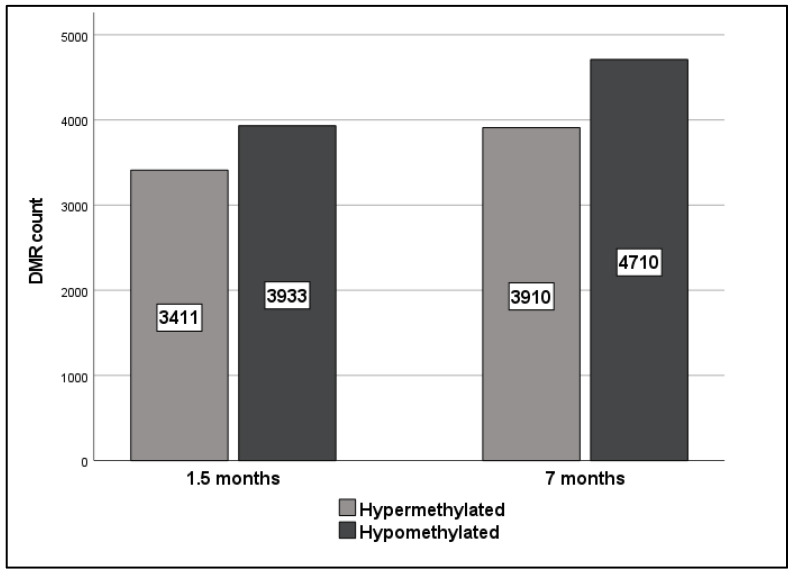
Significant hyper- (pale grey) and hypo- (dark grey) methylated DMR counts (*p*-value < 0.05) identified by SureSelect MethylSeq in rats receiving 27.6 Gy FI relative to sham-irradiated rats at 1.5 and 7 months after irradiation.

A total of 67,098 and 684,433 DMRs were identified across all chromosomes at 1.5 and 7 months after 27.6 Gy FI relative to sham-irradiated rats, respectively. After DMR filtering according to significance (*p*-value < 0.05), the number of DMRs dropped to 7344 and 8620 at 1.5 and 7 months, respectively (a detailed list of DMRs is provided in Supplementary Materials). Of those, 3933 and 4710 DMRs were hypomethylated while 3411 and 3910 DMRs were hypermethylated at 1.5 and 7 months after irradiation, respectively (Figure 2).

**Figure 3:**
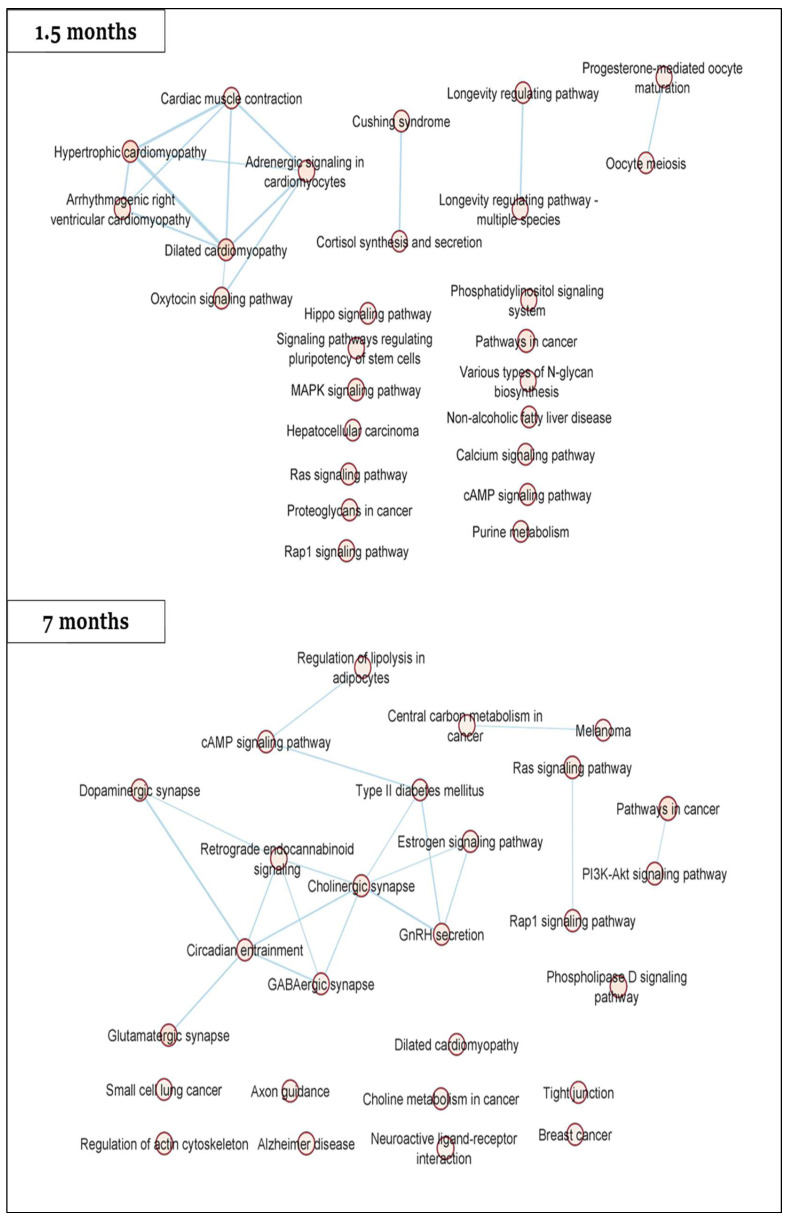
Pathway analysis of significant DMRs (*p*-value < 0.05). Statistically significant KEGG pathways (*p*-value < 0.05, Q-value < 0.25) were visualized by STRING-db and Cytoscape, respectively.

Pathway analysis of significant DMRs (*p*-value < 0.05) revealed the enrichment of several pathways, including the dilated cardiomyopathy pathway at both 1.5 and 7 months (Figure 3). Other cardiac relevant KEGG pathways were also enriched at 1.5 months (adrenergic signaling in cardiomyocytes, cardiac muscle contraction, hypertrophic cardiomyopathy, arrhythmogenic right ventricular cardiomyopathy, calcium signaling pathway and Hippo signaling pathway) as well as at 7 months (regulation of actin cytoskeleton and tight junction).

For downstream qPCR validation, cutoff criteria were applied yielding 10 and 24 DMRs at 1.5 and 7 months, respectively (detailed in Supplementary Materials). Next, the DMR list was further reduced by selection of DMRs previously linked to cardiovascular function in literature. A qPCR validation was performed for 8 DMRs (*SLMAP*, *LDLR*, *ITPR2*, *CDH18*, *CACNA1C*, *CELF4*, *E2F6* and *PTPN2)*. A brief description of these genes, as well as their connection to cardiovascular function/disease and observed methylation status, is provided in Table 1.

From STRING-db, multiple interactions were identified between dysregulated cardiac proteins and *LDLR* (calnexin), *ITPR2* (Phopholipase C beta3), *E2F* family (*RBBP7*) and *PTPN* family (*SLM2*, Thioredoxin, Ubiquiting-protein ligase B, galectin1, hrRNP K, cysteine and glycin-rich protein 1). In addition, two of the significantly dysregulated cardiac proteins were shown to interact with *CACNA1* family (Calsequestrin 2 and Myosin 4).

### 2.3. Hypomethylation of SLMAP at 1.5 Months Translates into a Dose-Dependent Increase in Gene Expression

Of the eight DMRs assessed by qPCR, only five genes were present in detectable quantities (*SLMAP*, *LDLR*, *ITPR2*, *E2F6* and *PTPN2*) (from now on called differentially methylated genes (DMGs)).

**Figure 4:**
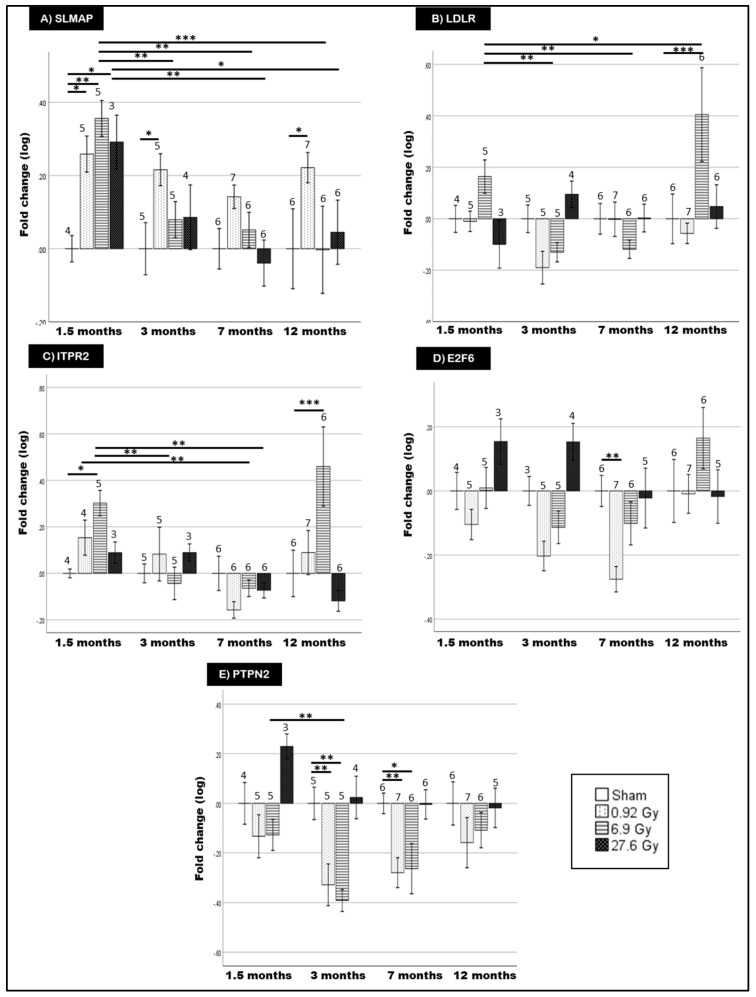
mRNA expression levels of *SLMAP* (**A**), *LDLR* (**B**), *ITPR2* (**C**), *E2F6* (**D**) and *PTPN2* (**E**) in the blood of rats receiving either sham irradiation (0 Gy) or fractionated irradiation of 0.92, 6.9 and 27.6 Gy and sampled after 1.5, 3, 7 and 12 months. Data are presented as log fold change normalized to *PHLPP1* (* = *p*-value < 0.05, ** < 0.01, *** < 0.001). Number of rats per group is indicated atop their respective bars. Plotted values represent group means ± standard error of mean (SEM). Statistical analysis was performed by SPSS General linear model and generalized linear models for data following normal and non-normal distribution, respectively. Multiple comparison correction was performed using Fisher’s LSD.

*SLMAP* expression was significantly increased after 1.5 months in all irradiated rats (Figure 4A). This increased expression follows the observed hypomethylation after 27.6 Gy FI (Table 1). Significantly increased *SLMAP* expression continues to 3 and 12 months after 0.92 Gy FI.

For the other genes, *LDLR*, *ITPR2*, *E2F6* and *PTPN2* (Figure 4B–E), a number of gene expression alterations were detected, yet without consistent trends across radiation doses or follow-up time. Moreover, qPCR results did not reproduce the observed methylation pattern.

Correlation between DMG expression and rat GLS measurements identified a strong correlation between *E2F6* expression and the GLS of rats receiving 27.6 Gy FI (r = |0.872|, *p*-value < 0.05).

### 2.4. Two of the Selected Rat DMGs Show Altered Expression in BC Patient Blood

**Figure 5:**
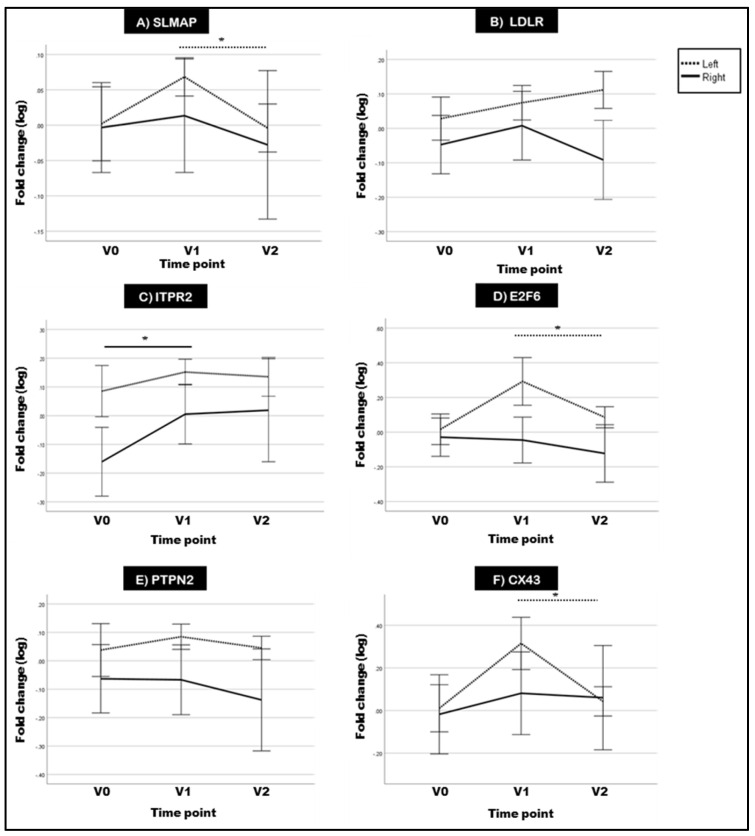
Mean log fold change of *SLMAP* (**A**), *LDLR* (**B**), *ITPR2* (**C**), *E2F6* (**D**), *PTPN* (**E**) and CX43 (*GJA1*) (**F**) expression in the blood of right- (n = 9) and left-sided (n = 16) BC patients sampled at diagnosis (V0), immediately after radiotherapy (V1) and 6 months after radiotherapy (V2). Data are presented as mean log fold changes in gene expression normalized to *TBP* ± SEM. Displayed significance values were calculated using observed log expression fold changes (* = *p*-value < 0.05). Statistical analysis was performed using SPSS generalized estimating equations module and multiple comparison correction was performed using LSD.

Patients of the MEDIRAD cohort with significantly higher cardiac radiation exposure showed a GLS-based subclinical left ventricular dysfunction (GLS decrease > 15%) 6 months after radiotherapy [33]. In the assayed BC patient blood, *SLMAP* showed a trend of increased expression for left-sided BC patients at V1 relative to V0, which decreases significantly at V2 (Figure 5A). Both *ITPR2* and *E2F6* showed increased expression at V1 relative to V0. However, the increase for *ITPR2* was only significant in right-sided BC patients, while for *E2F6* it was in left-sided BC patients (Figure 5C,D). On the other hand, LDLR and PTPN2 expression was unchanged (Figure 5B,E).

Previously, we demonstrated that selective inhibition of Connexin-43 (CX43) hemichannels alleviated radiation-induced endothelial cell damage [34]. In addition, *E2F6* was reported to affect the expression of CX43 gene (*GJA1*) in transgenic mice [31]. In our current experiments, we also found CX43 expression to be significantly increased at V1 in left-sided BC patient blood relative to V0 (Figure 5F).

In addition, patient stratification was performed according to whether the mean heart dose (MHD) was higher or lower than 2.5 Gy (Supplementary Figure S2). The 2.5 Gy MHD threshold was selected according to the German Society for Radiation Oncology (DEGRO) recommendations to minimize radiation-induced cardiotoxicity [35]. After stratification of data, the changes in *E2F6* and *SLMAP* were found to occur mainly at the higher radiation doses (>2.5 Gy). Correlation of MHD dose and gene expression indicated a medium correlation at V1 for *ITPR2*, *E2F6* and CX43 (*GJA1*) (*ITPR2*: r = |0.54|, *p*-value = 0.032; *E2F6*: r = |0.52|, *p*-value = 0.037; *GJA1*: r = |0.51|, *p*-value = 0.043) and at V2 for *SLMAP* (Pearson correlation coefficient r = |0.59|, *p*-value = 0.017).

## 3. Discussion

In the current study, we evaluated the effects of local heart irradiation on global and gene-specific DNA methylation in an experimental RICVD rat model. The validity of this model was previously confirmed by Ribeiro et al. who reported significant myocardial dysfunction (i.e., GLS decrease of >15%) at 12 and 18 months after 27.6 Gy FI [19]. Moreover, irradiated rats showed a decreased microvascular density (MVD) in apex of these rats’ hearts which has been proposed as a predictor of early left ventricular remodeling [19]. Previous research investigating radiation-induced global methylation effects has reported variable effects of hyper- and hypo-methylation [36,37,38], as well as an occasional absence of methylation effects [39,40,41]. Our results showed global hypomethylation at 12 months for all doses while a hypomethylation trend was observed over time after 0.92 and 6.9 Gy FI. However, interpretation of our global methylation results is difficult due to the high inter-replicate variability and low sample numbers in certain groups. Nevertheless, a strong association between global methylation and GLS levels was observed at 12 months after the 2 higher doses (6.9 and 27.6 Gy). The global hypomethylation observed after 27.6 Gy follows previous reports linking global DNA methylation with cardiac dysfunction [42,43,44,45]. As for gene-specific methylation, higher numbers of hypomethylated DMRs were found at both 1.5 and 7 months after 27.6 Gy FI. Although there are limited studies addressing the effects of ionizing radiation on gene-specific methylation, most of these studies declare a higher predilection to hypermethylation [4,46]. However, the direction of gene specific methylation appears to vary according to employed animal model/cell line and irradiation protocol [4].

Pathway analysis of significant DMRs revealed dilated cardiomyopathy as an enriched pathway in rat blood at both 1.5 and 7 months. Interestingly, Ribeiro et al. (MEDIRAD colleagues) performed a proteomic analysis on the rats’ cardiac tissues and also identified dilated cardiomyopathy as a significantly dysregulated protein pathway [19]. Other overlapping pathways between the rat DMRs (current study) and proteomics dataset [19] include cardiac muscle contraction, hypertrophic cardiomyopathy, adrenergic signaling in cardiomyocytes and longevity regulating pathway (DMRs at 1.5 months), as well as gonadotropin releasing hormone (GnRH) secretion, dopaminergic synapse, tight junction and circadian entrainment (DMRs at 7 months). All of these pathways have been previously implicated in cardiovascular impairment [47,48,49,50,51,52,53,54,55,56]. Dysregulation of these pathways has been known to result from oxidative stress, a proven contributor in radiation-induced cardiotoxicity [50,57,58,59,60,61,62]. Overall, pathway analyses of DMRs and proteomics datasets seem to support the occurrence of radiation-induced DNA methylation alterations in cardiac relevant genes which can affect functional protein levels.

Only *SLMAP* presented concurrent hypomethylation and overexpression at 1.5 months after 27.6 Gy FI. *SLMAP* represents a family of tail-anchored sarcolemmal membrane-associated proteins in the myocardium which regulates cardiac excitation-contraction [21,22,63]. Altered *SLMAP* methylation was previously documented in advanced atherosclerotic plaques of coronary heart disease patients [64]. *SLMAP* can also inhibit Hippo signaling; a DMR enriched pathway at 1.5 months was previously associated with dilated cardiomyopathy and ischemic heart disease [65]. Specifically, Hippo pathway activation induces DNA damage-induced cardiomyocyte apoptosis after irradiation which is particularly relevant in the ensuing cardiac toxicity [66,67]. Consequently, the observed hypomethylation and overexpression may serve as a protective mechanism against radiation-induced cardiac effects by promoting cardiac regeneration and cardiomyocyte proliferation. However, as *SLMAP* overexpression gradually decreases at the later time points, these protective effects seem to be limited by time.

For the other DMGs (*LDLR*, *ITPR2*, *E2F6* and *PTPN2*), there was poor correlation between methylation status and gene expression. This discordance could be due to the occurrence of the DMRs primarily in gene body locations (c.f. gene promoters). While the exact function of gene body DNA methylation is poorly understood, hypothesized functions include inhibition of alternative splicing [68] and prevention of transcription initiation at intergenic promoters [69]. Previous research has indicated a positive correlation between gene body methylation and gene expression [70,71]. However, other studies have also shown a negative relationship between gene body methylation and gene expression [72,73,74,75,76,77]. This could be due to gene body CpGs representing functional elements such as enhancers, alternative promoters, transcription factor binding sites, repetitive elements and enrichment of nucleosomes at intron-exon junctions [70,74]. Therefore, these observations suggest that DNA methylation’s regulation of gene expression is bidirectional with the location of CpG sites, disease context and relevant genes influencing the methylation effect [78].

Interestingly, Yao et al. previously reported the differential methylation of *E2F*, *PTPN* and *CDH* families in rat cardiac tissues after 6 months of acute 18 Gy of local heart irradiation [46]. As these rats also presented with RICVD, this suggests these gene families’ responsiveness to radiation-induced methylation alterations. In addition, *CACNA1C*, a DMR exhibiting >25% differential methylation at 1.5 and 7 months, was also differentially methylated in RICVD rats after acute 18 Gy irradiation [46]. Despite not being detectable by qPCR in our samples, *CACNA1C* was identified in a number of our enriched DMR pathways concurrently dysregulated in the cardiac proteome including dilated cardiomyopathy and hypertrophic cardiomyopathy [19]. The reproducible enrichment of *CACNA1C* points to a role for *CACNA1C* in myocardial dysfunction with extrapolated relevance in RICVD [79,80]. Therefore, further investigations of *CACNA1C*’s methylation status after FI and its involvement in RICVD are warranted. From predicted STRING protein-protein interactions (PPI), significantly dysregulated cardiac proteins in the irradiated rats [19] were shown to interact with *LDLR*, *ITPR2*, *E2F, PTPN* and *CACNA1* families, as well as the methylation relevant *DNMT3a* [81,82]. This points to a multi-dimensional regulation of cardiac responses to ionizing radiation.

Finally, selected DMGs were explored in the blood of BC patients treated with adjuvant radiotherapy. *SLMAP* expression tended to increase at V1 compared to V0 in left-sided BC patients. Statistical significance was not reached, possibly due to the limited sample numbers and high inter-individual variation of DNA methylation, especially in blood [83,84,85,86]. Interestingly, after an initial increase at V1, *SLMAP* expression decreased at 6 months after radiotherapy (V2) in irradiated left-sided BC patients while presenting a medium correlation with MHD at V2. A similar initial *SLMAP* upregulation, which gradually decreases over time, was also observed in the irradiated rats. As decreased expression of *SLMAP* was found in human dilated ventricles, the observed *SLMAP* downregulation over time could contribute to cardiac dysfunction [22,87]. Interestingly, decreased *SLMAP* protein levels were also observed in cardiac tissues of Mayak workers diagnosed with ischemic heart diseases after occupational exposure to >500 mGy external gamma rays [88], which further supports the possible involvement of *SLMAP* in RICVD.

*ITPR2* is the major cardiac isoform of a family of calcium channels whereby increased *ITPR2* expression activates calcium dependent signaling and modulates excitation-contraction coupling in cardiomyocytes [25]. In addition, *ITPR2* overexpression has been linked to many cardiac pathologies including cardiac arrhythmias, failure and hypertrophy [25,89,90,91]. In our study, hypermethylation was associated with an increased expression of *ITPR2* for the 6.9 Gy FI dose at 1.5 and 12 months after irradiation in rats. In humans, right-sided BC patients showed significantly higher *ITPR2* expression at V1 relative to V0. Consequently, *ITPR2* dysregulation seems to occur as a result of radiation in both rats and BC patients.

*E2F6* is a member of the *E2F* family that functions as a transcriptional repressor [92]. In-vivo, forced *E2F6* overexpression was associated with cardiac remodeling and dilated cardiomyopathy [31,93]. In addition, pathway analysis of Mayak nuclear workers’ cardiac tissue proteomes showed that *E2F* family was dysregulated in irradiated groups compared to controls [94]. Our findings show *E2F6* hypomethylation at 1.5 months after 27.6 Gy FI in rats with variable expression and a seemingly dose-differential effect whereby low doses induce *E2F6* downregulation (c.f. 0.92 Gy), as shown in mouse embryos exposed to low-dose X-rays [95]. In addition, the strong correlation between *E2F6* and GLS alterations after 27.6 Gy FI suggests a possible contribution to the observed myocardial dysfunction. *E2F6* also exhibits significantly higher expression at V1 relative to V0 in left-sided patients. Stratification of patients, according to the received mean heart dose (MHD), showed *E2F6* dysregulation at higher MHDs (>2.5 Gy) in left-sided BC radiotherapy patients while maintaining a medium correlation to MHD. This further strengthens the potential involvement of *E2F6* in developing radiation-induced cardiac effects. However, further investigations in larger cohorts could help characterize the functional impact.

Finally, Connexin-43 (CX43) is a transmembrane protein forming gap junctions and hemichannels which are involved in intercellular communication [96]. CX43 was reported to increase the formation of atherosclerotic lesions in vivo [97,98]. We previously reported that single and fractionated X-irradiation induced an acute and persistent increase in CX43 gene and protein levels in human endothelial cells while selective inhibition of CX43 hemichannels alleviated radiation-induced endothelial cell damage [34,99]. In the current study, CX43 expression was significantly increased at V1 in the blood of left-sided BC patients relative to V0, in a similar manner to *E2F6*. Therefore, further investigation into the relationship between *E2F6* and CX43 in the scope of radiation-induced cardiovascular dysfunction is needed.

### Study Limitations

Despite having the advantages of offering long-term longitudinal follow-up of identified DMRs over time, our study has a number of limitations. (1) Our first sampling time point for the rats was 1.5 months after irradiation. Consequently, we are unable to comment on any methylation alterations occurring at earlier time points. (2) Methylation analysis was performed in peripheral blood which introduces the confounder of different methylation profiles due to differing blood cell fraction counts [100,101,102]. DNA in blood is a mixture of DNA from blood cells and circulating cell-free DNA released from dying cells [102,103,104]. Local heart irradiation, as in our experimental rat irradiation model, primarily affects the methylation of the heart, as well as circulating blood cells. However, considering the short lifespan of circulating blood cells, delayed methylation alterations are most likely not the result of irradiated blood cells [105]. In addition, peripheral blood/leucocyte fraction methylation patterns have been frequently employed in DNA methylation biomarker research for cardiovascular disease identifying associations near genes unrelated to immune function or inflammation [106,107,108]. This supports the usefulness of blood-based DNA methylation investigations despite confounders, especially when considering the convenience of blood as a sample source. (3) The number of available samples was limited for certain rat sampling time points/doses due to technical limitations. (4) The primary validation in BC patients treated with adjuvant radiotherapy involved a somewhat limited number of patients (right-sided patients (n = 9) and left-sided patients (n = 16)) which necessitates confirmation in bigger patient cohorts.

## 4. Materials and Methods

### 4.1. Animals and Irradiation

Adult female Wistar rats (12–14 weeks old) underwent whole heart X-irradiation of 0.04, 0.3 and 1.2 Gy for 23 consecutive days (weekend excluded), resulting in cumulative doses of 0.92, 6.9 or 27.6 Gy. Control rats were sham-irradiated [0.0 Gy) following the same procedure. This translational experimental model was performed at MEDIRAD consortium partner, Centro Cardiovascular da Universidade de Lisboa (CCUL) [19]. There, blood was collected at 1.5, 3, 7 and 12 months after irradiation. Blood samples were received on dry ice and stored at −80 °C until further processing.

### 4.2. DNA Extraction

DNA was extracted from 200 µL frozen blood pellets using QIAamp DNA mini kit (Qiagen, Hilden, Germany) according to kit protocol. However, the extracted DNA concentration was found to be low [1–5 ng/µL). To increase the efficiency of DNA extraction, phenol/chloroform/isoamyl alcohol mixture [25:24:1 PCI) was incorporated into the DNA extraction protocol. Briefly, after sample thawing, samples were incubated with proteinase K and buffer AL at 56 °C for 10 min. 700 µL PCI was added per sample and mixed for 1.5 h at 1400 rpm at room temperature (Eppendorf Thermomixer C, Eppendorf AG, Hamburg, Germany). Next, samples were centrifuged for 5 min at 14,000× *g*. The upper aqueous layer containing the DNA was collected and 1–1.5 mL 100% ethanol as well as 50 µL buffer AL were added to precipitate the DNA. Finally, this mixture was transferred to QIAamp DNA mini kit column in 700 µL aliquots. Extraction was continued using QIAamp DNA mini kit following manufacturer recommendations. DNA concentration and purity were determined by comparing the ratio of optical density (OD) at 260 and 280 nm.

### 4.3. Global DNA Methylation Using MethylFlash Global DNA Methylation

Absolute global 5-methyl cytosine (5 mC) levels were analyzed in extracted DNA using MethylFlash Global DNA Methylation (5 mC) ELISA Easy Kit (Epigentek Group Inc., Farmingdale, NY, USA) according to manufacturer protocol. The kit measures 5 mC content as a percentage of total cytosine content. An amount of 100 ng of purified DNA was added to the ELISA plate. The methylated fraction of DNA was detected using 5 mC specific antibodies and quantified colorimetrically by measuring OD at 450 nm. The positive control (PC) supplied with the kit was used to generate a standard curve. The slope of the standard curve was calculated and used to determine the concentration of 5 mC in the samples as follows: 5 mC% = [(Sample OD − Negative control OD)/(Slope * DNA quantity)] × 100.

### 4.4. Gene-Specific DNA Methylation Analysis Using SureSelect Methyl-Seq

Gene-specific methylation analysis was performed with the Rat SureSelect Methyl-Seq platform (Agilent Technologies Inc., Santa Clara, CA, USA). SureSelect MethylSeq is a type of methylation capture sequencing (MC-seq) using biotinylated RNA baits to capture the genomic areas of interest for subsequent bisulfite sequencing. This method allows quantitative analysis of DNA methylation with single base resolution [109,110]. The rat SureSelect MethylSeq has been designed to target non-redundant promoters, CpG islands, island shores as well as previously identified GC-rich sequences [111]. Sixteen samples were selected to undergo SureSelect Methyl-Seq library preparation: sham-irradiated rats at 1.5 months (n = 4), 27.6 Gy irradiated rats at 1.5 months (n = 4), sham-irradiated rats at 7 months (n = 4) and 27.6 Gy irradiated rats at 7 months (n = 4). Due to technical limitations which necessitated high DNA sample concentrations [3 μg) with limited available blood per rat, sample size per group was limited. Library preparation and sequencing were performed in collaboration with the Ghent University sequencing facility NXTGNT (Ghent, Belgium) and GENEWIZ global genomics service company (GENEWIZ Germany GmbH, Leipzig, Germany). Library preparation, probe-based target enrichment, bisulfite treatment, and library indexing PCR were performed according to SureSelect^XT^ Methyl-Seq Library Preparation kit (Agilent Technologies Inc., Santa Clara, CA, USA) protocol (Version E0, April 2018). The libraries were equimolarly pooled and sequenced together with a 20% PhiX control spike-in v3 on Illumina Hiseq 4000 (Illumina, Inc., San Diego, CA, USA), generating approximately 1.2 × 10^9^ paired-end reads of 150 base pair length.

For the sequencing data analysis, similar methods were applied as was previously described [112]. Raw-read quality control was assessed using FASTQC (version 11.9). This was followed by reads trimming using Trim Galore (version 0.6.4) with the paired-end mode using the default parameters. Reads quality post trimming was reassessed as well (using FASTQC). Using bismark (version 0.19.0), reads were mapped to *Rattus norvegicus* genome which utilizes bowtie 2 (version 2.3.3), with a maximum of 1 mismatch in the seed region. The *Rattus norvegicus* reference genome (Rnor6.0) was downloaded from ftp://ftp.hgsc.bcm.edu/Rnorvegicus/Rnor6.0/, on 15 January 2020, and then indexed using Bismark with *bismark_genome_preparation* script. After mapping, these temporary changes were reverted. Subsequently, PCR duplications were removed using *deduplicate_bismark* script and a post-alignment quality control was performed using *flagstat* option of samtools (version 1.6) and *stats* option of BamUtil (version 1.0.14). The methylation level was assessed for each methylation context separately (for cytosines followed by guanines (CpGs), non-guanines and guanines (CHGs), two non-guanines (CHHs) or any other possibilities (CNs)). This was executed using *bismark_methylation_extractor* with the following flags: paired-end, no-overlap, and minimum coverage of at least 1 read, whilst the remaining parameters were set to the default settings.

For the rest of the analysis, only CpG methylations were included. For this, BSseq package (version 1.18.0) was used in Bioconductor. First, the data were smoothed using *BSmooth* function allowing 20 CpGs as a minimum within a window of 500, thereby smoothing the methylation levels across the CpGs within that window. This was used to establish thresholds for t-statistics (calculated using BSmooth.tstat function) across the groups using 1st and 99th quantile percentiles. Only CpGs with a minimum coverage of 10× within at least 3 samples were retained, and differentially methylation regions (DMRs) were identified using *dmrFinder* command. Each identified DMR was subjected to 1000 iterations of permutations (with randomization) that re-calculate the t-statistics for each permutation, and *p*-values were calculated and corrected using Benjamini-Hochberg false discovery rates (FDRs), for multiplicity problem. The *p*-values were calculated as the fraction of null areas (retrieved after each permutation) exceeding the observed area (before permutation). This was executed twice, performing pairwise comparison between sham-irradiated vs. 27.6 Gy at 1.5 and 7 months, separately. After that, DMRs were annotated to the rat genome (assembly Rnor_6.0) using closest from bedtools.

### 4.5. Pathway Analysis of Rat Differentially Methylated Regions (DMRs) by STRING-db

Pathway analysis of significant SureSelect MethylSeq DMRs (*p*-value < 0.05) at 1.5 and 7 months after 27.6 Gy or 0 Gy (sham) was performed using the STRING database (V.11.2). STRING is a database dedicated to organism-wide protein association networks by integrating known and predicted associations between proteins, including both physical interactions and functional associations [113]. The produced protein-protein interaction (PPI) network was then exported to Cytoscape 3.9.0 [114] where STRING enrichment was retrieved and enrichment maps were constructed using *EnrichmentMap* app in Cytoscape (V.3.3.3).

### 4.6. Investigation of Expression Alterations in DMRs Using Quantitative PCR

Validation of the rat DMRs was performed by quantitative Real Time PCR (qRT-PCR) in the blood of rats irradiated with 0, 0.92, 6.9 and 27.6 Gy FI at 1.5, 3, 7 and 12 months. Selection of the “top” target genes was performed by filtering the SureSelect MethylSeq output to show only DMRs with significant (*p*-value < 0.05) methylation difference (>25%) to limit downstream analyses [115,116,117]. Afterwards, a literature search of the filtered DMRs was performed to focus on genes with documented association to cardiovascular disease. This led to 8 selected genes: *SLMAP*, *LDLR*, *ITPR2*, *CDH18*, *CACNA1C*, *CELF4*, *E2F6* and *PTPN2*. Only *SLMAP*, *LDLR*, *ITPR2*, *E2F6* and *PTPN2* were detectable in rat blood.

RNA was extracted from frozen rat blood using NucleoSpin RNA Blood Mini kit (Macherey-Nagel GmbH & Co. KG, Düren, Germany) according to manufacturer instructions. Then, reverse transcription of extracted RNA was performed using GoScript Reverse Transcription Mix employing random primers (Promega Corporation, Madison, WI, USA). Four genes were assayed as reference genes (*POLR2A*, *TBP*, *ACTB* and *PHLPP1*). Selection of the reference gene was performed using NormFinder [118] whereby *PHLPP1* showed the highest stability and was selected for normalization. The expression levels of selected DMR transcripts were determined by qPCR using TaqMan Gene Expression Assays (Thermo Fisher Scientific, Waltham, MA, USA) (*SLMAP*: Rn01401804_m1; *LDLR*: Rn00598442_m1; *ITPR2*: Rn00579067_m1; *E2F6*: Rn01499181_m1; *PTPN2*: Rn00588846_m1; *PHLPP1*: Rn00572211_m1). Next, qPCRs were performed using Fast Advanced Master Mix (Thermo Fisher Scientific, Waltham, MA, USA) on qTOWER^3^ touch thermal cycler (Analytik Jena, Jena, Germany). Relative quantification was calculated using the equation log2^−ΔΔCT^, where ΔΔC_T_ = [C_T_ of target gene − C_T_ of reference gene]_irradiated group_ − [C_T_ of target gene − C_T_ of reference gene]_sham group_.

Significantly dysregulated proteins in cardiac tissues of rats receiving 27.6 Gy FI, supplied by MEDIRAD consortium partners [19], were queried in STRING-db (showing 50 interactors in 1st and 2nd shell) to reveal any relevant interactions with the rat DMGs.

### 4.7. Correlation of Rat Global DNA Methylation and DMR Expression with Global Longitudinal Strain (GLS)

MEDIRAD consortium partner, CCUL, evaluated the cardiac function of the irradiated rats and reported a dose-dependent reduction in GLS (>15%) [19]. 5 mC% levels and qPCR expression levels of rat DMGs were correlated with GLS in rats sacrificed at 12 months after irradiation.

### 4.8. Investigating Gene Expression of Selected DMRs in Breast Cancer Patients’ Blood

#### 4.8.1. Patient Selection

Blood pellets were collected from MEDIRAD EARLY HEART cohort of BC patients treated with adjuvant radiotherapy. This was performed at our consortium partner University Medical Center Groningen (UMCG), including a random selection of 25 BC patients with right-sided (n = 9) and left-sided BC (n = 16) [33]. The study was approved by the Ethics committee at UMCG (NL62360.042.17). Female unilateral BC patients aged 40–75 years treated with primary breast conserving surgery and postoperative radiotherapy were recruited during their first visit with the radiation oncologist. All patients signed a written informed consent form. Patients with previous medical history of coronary artery disease and/or myocardial infarction and/or atrial fibrillation were excluded. The patients were classified as having left- or right-sided BC according to the anatomical position of the tumor.

#### 4.8.2. Radiotherapy Protocol

The total dose for the breast was 40.05–43.6 Gy. This dose was delivered in 15–20 separate fractions with a volumetric modulated arc therapy/fixed-field intensity-modulated radiotherapy (VMAT/IMRT) technique. Left-sided BC patients were treated with deep inspiration breath hold using the active breathing control system in order to lower the cardiac dose as much as possible.

#### 4.8.3. Blood Collection and Reverse Transcription qPCR

Blood was collected from the patients in EDTA vacutainers at three time points: at diagnosis (V0), directly after radiotherapy (V1) and 6 months after radiotherapy (V2). Blood samples were centrifuged at 1500× *g* for 15 min to separate the plasma. Blood pellets were then stored at −80 °C until further processing. RNA extraction, cDNA synthesis and gene expression analysis were performed using the same protocols detailed for the rat samples. TaqMan real-time PCR assays were used for qPCR quantification of *SLMAP*, *LDLR*, *ITPR2*, *E2F6*, *PTPN2* expression relative to reference gene, *TBP* (*SLMAP*: Hs01058330_g1; *LDLR*: Hs00181192_m1; *ITPR2*: Hs00181916_m1; *E2F6*: Hs01034552_m1; *PTPN2*: Hs00959888_g1; *TBP*: Hs00427620_m1).

### 4.9. Statistical Analysis

Normality of all datasets was assessed by Shapiro-Wilk test. Analysis of global methylation was performed using a generalized linear model with least significant difference (LSD) correction for multiple comparisons. Analysis of normally distributed parametric rat qPCR data was performed using a general linear model with LSD correction for multiple comparisons. For non-parametric rat qPCR data, analysis was performed using a generalized linear model with LSD correction for multiple comparisons. Correlation with functional data was performed by calculating Pearson correlation coefficient. Statistical analysis of BC patient qPCR data was performed using a generalized estimating equation to accommodate for the nonparametric characteristics of the data. All detailed statistical analyses were performed using SPSS version 28 (IBM Corp., Armonk, NY, USA).

## 5. Conclusions

The involvement of DNA methylation alterations in RICVD pathogenesis is underexplored. In the current study, we attempted to identify DNA methylation alterations related to rat whole-heart irradiation. The highest dose of radiation (27.6 Gy FI) resulted in blood DMRs associated with multiple cardiac relevant pathways including dilated cardiomyopathy and hypertrophic cardiomyopathy. This suggests the involvement of DNA methylation alterations in the onset of myocardial dysfunction. The expression of selected DMRs (significant differential methylation >25% with cardiovascular relevance) was assayed and discordance between methylation-predicted expression and observed expression suggests that gene body DNA methylation regulates gene expression in a multi-factorial bidirectional manner. *SLMAP*, *ITPR2*, *E2F6* and *PTPN2* showed differential methylation and expression in irradiated rats, while *E2F6* expression correlated with GLS measurements at 12 months after 27.6 Gy FI. Three of these rat DMGs (*SLMAP*, *ITPR2* and *E2F6*) also exhibited altered expression in BC patient blood, of which *SLMAP* and *E2F6* overexpression occurs mainly at higher MHDs. While this study provides some preliminary insights into radiation-induced DNA methylation alterations and their possible contribution to RICVD, further mechanistic validation by gene knockout/overexpression experiments, as well as large scale clinical studies are needed to validate their connections to RICVD.

## Figures and Tables

**Table 1:** DMRs selected for downstream validation, their correlation to cardiovascular function/disease and their altered methylation status after 27.6 Gy FI.

DMR	Connection to Cardiac Function/Disease	Methylation State after 27.6 Gy FI Dose Relative to Sham Irradiated Rats (*p*-Value < 0.05)
*SLMAP* *(Sarcolemma Associated Protein)*	*SLMAP* is a component of cardiac membranes involved in excitation-contraction (E-C) coupling and its perturbation results in progressive deterioration of cardiac electrophysiology and function [21].	Hypomethylated at 1.5 months after irradiation
	*SLMAP* also interacts with cardiac myosin suggesting a direct role in controlling cardiomyocyte contraction [22].	
*LDLR* *(Low Density Lipoprotein Receptor)*	Knockouts and/or mutations in *LDLR* lead to ineffective clearance of serum low density lipoprotein (LDL) cholesterol and contribute to premature atherosclerosis and cardiovascular disease [23].	Hypomethylated at 7 months after irradiation
*ITPR2* *(Inositol 1,4,5-Trisphosphate Receptor Type 2)*	Certain polymorphs of *ITPR2* have been associated with higher systolic blood pressure. *ITPR2* is expressed widely in myocytes with altered expression in heart failure [24,25].	Hypomethylated at 7 months after irradiation
*CDH18* *(Cadherin 18)*	A deletion involving *CDH18* was reported to be found in a case of congenital heart disease [26].	Hypomethylated at 1.5 months after irradiation
	In a study involving copy-number variants and the risk of sporadic congenital heart disease, rare deletions in study participants with congenital heart disease were in found in a number of genes including *CDH18* [27].	
*CACNA1C* *(Calcium Voltage-Gated Channel Subunit Alpha1 C)*	*CACNA1C* is a part of voltage-gated L-type calcium channel gene which plays an important role in cardiac electrical excitation [28].	Hypomethylated at 1.5 and 7 months after irradiation
*CELF4* *(CUGBP Elav-like family member 4)*	A polymorphism of *CELF4* has been reported to have a modifying effect on anthracycline-related cardiomyopathy [29].	Hypomethylated at 7 months after irradiation
*E2F6* *(E2F Transcription Factor 6)*	*E2F6* is a cell cycle regulator, abrogation of expression of *E2F6* in neonatal cardiac myocytes leads to a significant decrease in myocyte viability suggesting a role in myocardial regeneration [30,31].	Hypomethylated at 1.5 months after irradiation
	Forced *E2F6* expression activates gene expression in myocardium resulting in dilated cardiomyopathy [31].	
*PTPN2* *(Protein Tyrosine Phosphatase Non-Receptor Type 2)*	Decreased expression of *PTPN2* through activation of miR-201 leads to attenuation of apoptosis and improvement of migration of cardiac stem cells exposed to hypoxia which would in turn increases their potential to repair the injured myocardium [32].	Hypomethylated at 7 months after irradiation

## Data Availability

The SureSelect methylation data presented in this study have been accessioned in the Sequence Read Archive (http://www.ncbi.nlm.nih.gov/sra) under BioProject Accession Number PRJNA808832.

## Supplementary Materials

The following supporting information can be downloaded at: https://www.mdpi.com/1422-0067/23/24/16214/s1.
