# Correction: Sallam et al. DNA Methylation Alterations in Fractionally Irradiated Rats and Breast Cancer Patients Receiving Radiotherapy. *Int. J. Mol. Sci.* 2022, *23*, 16214

**DOI:** 10.3390/ijms242417590

**Published:** 2023-12-18

**Authors:** Magy Sallam, Mohamed Mysara, Mohammed Abderrafi Benotmane, Radia Tamarat, Susana Constantino Rosa Santos, Anne P. G. Crijns, Daan Spoor, Filip Van Nieuwerburgh, Dieter Deforce, Sarah Baatout, Pieter-Jan Guns, An Aerts, Raghda Ramadan

**Affiliations:** 1 Radiobiology Unit, Interdisciplinary Biosciences, Belgian Nuclear Research Centre, SCK CEN, 2400 Mol, Belgium; 2 Laboratory of Physiopharmacology, University of Antwerp, 2610 Wilrijk, Belgium; 3 Institut de Radioprotection et de Sureté Nucléaire (IRSN), PRP-HOM, SRBE, LR2I, 92260 Fontenay-aux-Roses, France; 4 Centro Cardiovascular da Universidade de Lisboa (CCUL@RISE), Lisbon School of Medicine of the Universidade de Lisboa, 1649-028 Lisbon, Portugal; 5 Department of Radiation Oncology, University Medical Center Groningen, University of Groningen, 9713 GZ Groningen, The Netherlands; 6 Laboratory of Pharmaceutical Biotechnology, Ghent University, 9000 Ghent, Belgium; 7 Department of Molecular Biotechnology, Ghent University, 9000 Ghent, Belgium

Radia Tamarat and Susana Constantino Rosa Santos were not included as authors in the original publication [1]. These authors contributed to the published research article by performing rat heart irradiation and blood sampling experiments. These authors were missing from the published version due to an accidental editorial mistake made by the authors. The corrected Author Contributions statement appears here. The authors state that the scientific conclusions are unaffected. This correction was approved by the Academic Editor. The original publication has also been updated.
