# Supplementary material for: DNA Methylation Alterations in Fractionally Irradiated Rats and Breast Cancer Patients Receiving Radiotherapy
Source: Int J Mol Sci. 2022 Dec 19;23(24):16214. doi: 10.3390/ijms232416214 (PMC9783664; doi:10.3390/ijms232416214)
Supplement: Supplementary file 1 [file ijms-23-16214-s001.zip › ijms-2079750-supplementary/final supplementary figures.pdf]

Supplementary Figures

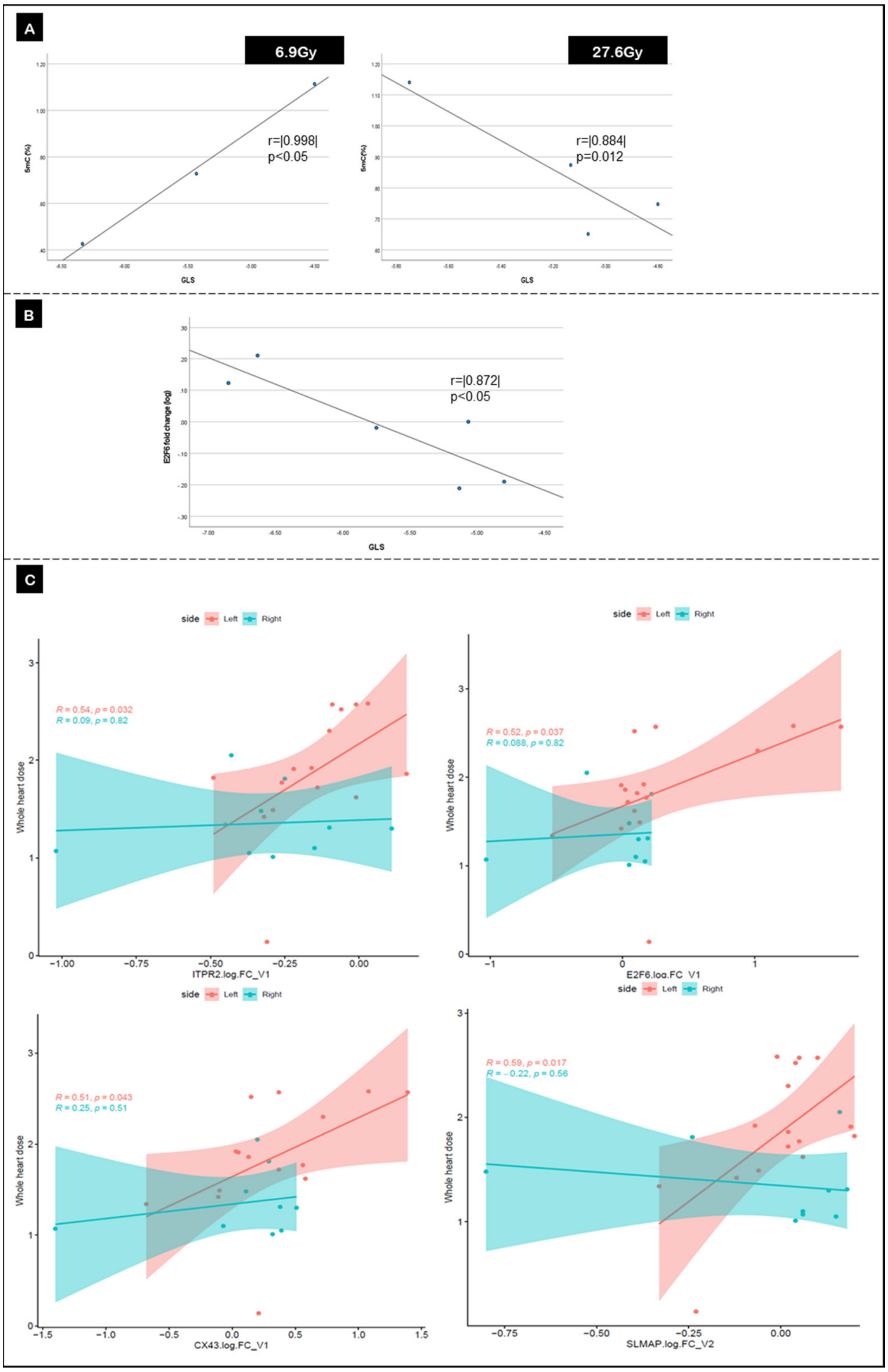

Supplementary Figure S1. Summary of relevant correlations A) Correlation between global methylation (5mC%) and GLS in rats receiving 6.9 and 27.6Gy FI. B) Correlation between E2F6 fold change (log) and GLS in rats receiving 27.6Gy FI. C) Correlation between V1 ITPR2, E2f6 and CX43 (upper left, upper right and lower left, respectively) and V2 SLMAP (lower right) fold change (log) and whole heart dose. Pearson correlation was calculated using SPSS version 28 and R program ggscatter function.

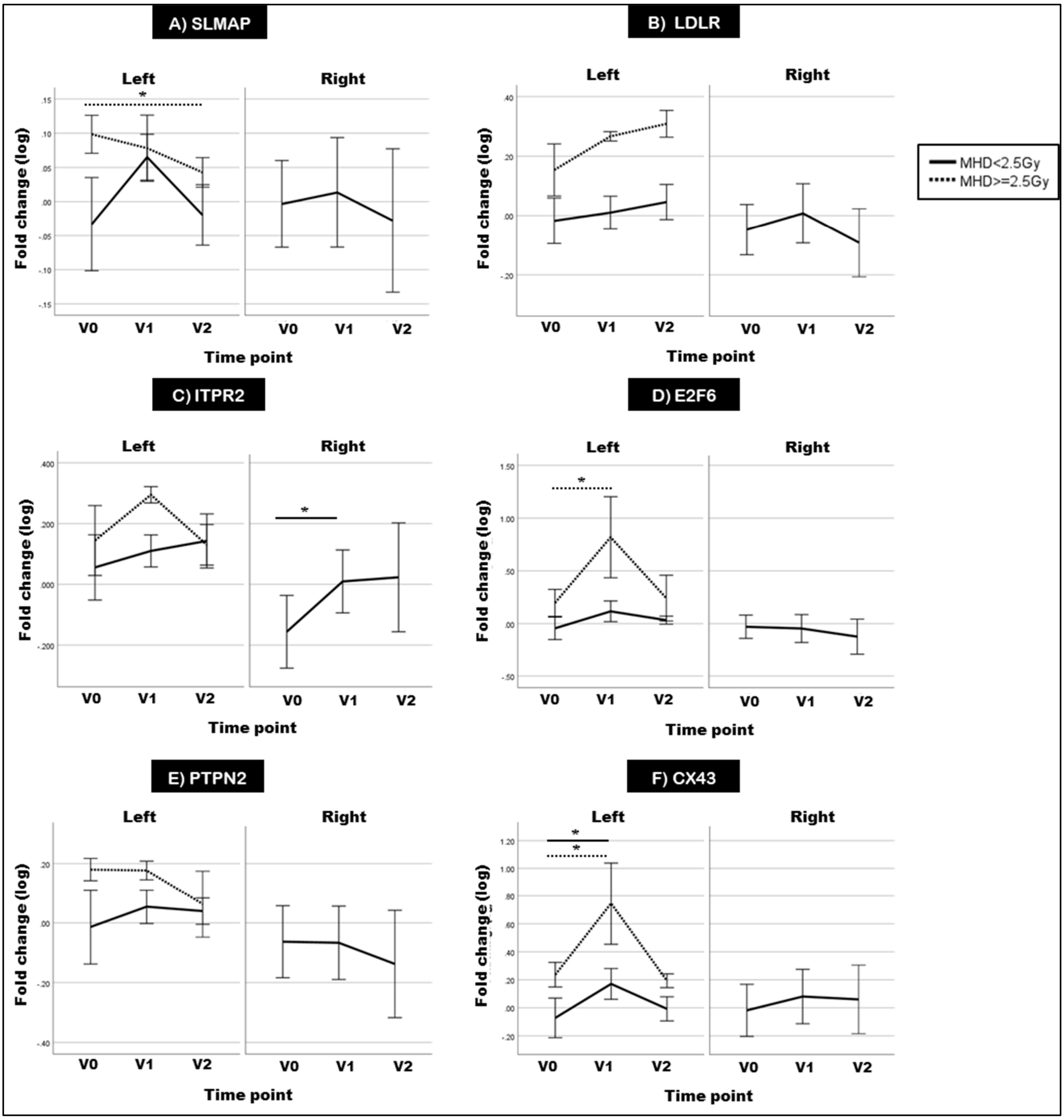

Supplementary Figure S2. Mean log fold change of SLMAP, LDLR, ITPR2, E2F6, PTPN and CX43 expression in the blood of right (n=9) and left sided ((MHD >2.5 Gy, n=4), (MHD<2.5 Gy, n=12)) breast cancer patients sampled at diagnosis (V0), immediately after radiotherapy (V1) and 6 months after radiotherapy (V2). Data is presented as mean log fold changes normalized to TBP ± standard error of mean. Continuous lines represent the mean log fold changes of patients who received <2.5 Gy mean heart dose (MHD) and dotted lines represent patients who received >= 2.5 Gy MHD..(*=p<0.05). Statistical analysis was performed using SPSS generalized estimating equations module and multiple comparison correction was performed using least significant difference (LSD).
